# Associations between Trunk Extension Endurance and Isolated Lumbar Extension Strength in Both Asymptomatic Participants and Those with Chronic Low Back Pain

**DOI:** 10.3390/healthcare4030070

**Published:** 2016-09-19

**Authors:** Rebecca Conway, Jessica Behennah, James Fisher, Neil Osborne, James Steele

**Affiliations:** 1 School of Sport, Health and Social Sciences, Southampton Solent University, East Park Terrace, Southampton SO14 0YN, UK; 0conwr69@solent.ac.uk (R.C.); 0behej96@solent.ac.uk (J.B.); james.fisher@solent.ac.uk (J.F.); 2 AECC Clinic, Anglo European College of Chiropractic, Bournemouth BH5 2DF, UK; NOsborne@aecc.ac.uk

**Keywords:** strength, endurance, isolated lumbar extension, trunk extension, low back pain

## Abstract

*Background:* Strength and endurance tests are important for both clinical practice and research due to the key role they play in musculoskeletal function. In particular, deconditioning of the lumbar extensor musculature has been associated with low back pain (LBP). Due to the relationship between strength and absolute endurance, it is possible that trunk extension (TEX) endurance tests could provide a proxy measure of isolated lumbar extension (ILEX) strength and thus represent a simple, practical alternative to ILEX measurements. Though, the comparability of TEX endurance and ILEX strength is presently unclear and so the aim of the present study was to examine this relationship. *Methods:* Thirty eight healthy participants and nineteen participants with non-specific chronic LBP and no previous lumbar surgery participated in this cross-sectional study design. TEX endurance was measured using the Biering–Sorensen test. A maximal ILEX strength test was performed on the MedX lumbar-extension machine. *Results:* A Pearson’s correlation revealed no relationship between TEX endurance and ILEX strength in the combined group (*r* = 0.035, *p* = 0.793), the chronic LBP group (*r* = 0.120, *p* = 0.623) or the asymptomatic group (*r* = −0.060, *p* = 0.720). *Conclusions:* The results suggest that TEX is not a good indicator of ILEX and cannot be used to infer results regarding ILEX strength. However, a combination of TEX and ILEX interpreted together likely offers the greatest and most comprehensive information regarding lumbo-pelvic function during extension.

## 1. Introduction

Low back pain (LBP) is one of the most common medical disorders in today’s society [1], affecting both the Western developed world [2] and less economically developed countries [3,4,5,6,7]. This results in a considerable economic burden worldwide and includes the costs of healthcare, indemnity payment, staff training and productivity loss [8,9]. With over 100 million work days lost per year in the UK [10] and an estimated yearly total cost of £10.6 billion [11], LBP not only affects the individual, but places a great strain on families, industries and governments [3,12]. A further financial strain is also placed on institutions as accurate clinical assessments of musculoskeletal function often rely on specialist, expensive equipment.

An estimated 60%–80% of the population report back pain at some time in their life, with it often being recurrent and persistent [13]. However, only 5%–15% of this population have attributed LBP to a specific cause. The remaining 85% cannot be given a precise patho-anatomical diagnosis, and so is referred to as “non-specific” LBP [14]. Although many sufferers will recover in the acute stages without intervention, approximately 40% of individuals will develop chronic LBP [15]. This is defined as pain in the area between the inferior margin of the twelfth rib and the inferior gluteal fold [3,16], with the duration of symptoms longer than twelve weeks [17,18].

Due to the symptoms experienced, people suffering with chronic LBP tend to restrict movement, avoiding using their low back in everyday situations because of fear of pain [19]. Research suggests that this reduction in physical activity may cause muscle atrophy, as a result of disuse of the lumbar extensors [20]. However, several researchers have reported no difference in physical activity levels of chronic LBP participants compared with asymptomatic controls [21,22,23]. Therefore, the relationship may in fact be bidirectional, in which deconditioning itself is seen as a factor contributing to the intolerance of physical activity [24]. Whichever the case, one of the multi-factorial dysfunctions consistently reported in the literature is the deconditioning of the lumbar extensor musculature, i.e., thoracic and lumbar erector spinae, multifidus and quadratus lumborum [25,26,27]. One role of the lumbar extensor musculature is to provide stability to the lumbar spine [28]. As such deconditioning in these muscles has been speculated to lead to spinal instability and contribute to the high recurrence rate in CLBP (chronic low back pain) [29].

This diminished function has invited research into the association between back strength and endurance and LBP. Numerous testing methods exist to examine the function of the lumbo-pelvic complex in extension. Broadly though they can be generalised into tests to either examine trunk extension (TEX) or isolated lumbar extension (ILEX).

During dynamic actions, TEX utilises both the paraspinal muscle group and the hip extensors in order to extend the low back and the pelvis through a range of motion (ROM) of approximately 180 degrees [30]. ILEX function, which isolates the lower back muscles through the prevention of pelvic rotation, is responsible for a ROM of approximately 72 degrees [31]. Therefore, TEX might best be considered a compound movement that results in additional backward rotation of the pelvis as the hip extensors contract. As a result, it is not possible to conclude specifically whether differences occur due to contribution from the hip extensors or the lumbar extensors. In contrast, since ILEX involves stabilising the pelvis to isolate the lumbar spine and remove torque produced by the hip extensors, this allows for a more precise measurement of the smaller and weaker lumbar extensor muscles [32]. 

However, as it does at least partly involve contributions from the paraspinal musculature, and that there is a well evidenced relationship between muscular strength and absolute muscular endurance [33], results obtained from TEX endurance methods of testing may be potential indicators of lumbar extension strength. As such these tests could be utilised in both prospective studies of risk factors for LBP in addition to cross sectional studies. In previous research, a number of studies implementing the Biering-Sorensen test as a measure of TEX endurance have reported significantly reduced holding times in the CLBP groups [19,34,35,36,37,38,39,40]. This suggests that chronic LBP is associated with decreased endurance of the trunk extensor muscles. In addition, studies implementing ILEX methods of testing have also reported chronic LBP participants to be significantly weaker than asymptomatic participants [26,41,42,43,44,45]. Therefore, the aim of this study was to investigate whether there is a relationship between TEX and ILEX to determine the necessity of adequate pelvic restraints. A correlation between the two methods would suggest that TEX could in fact be conducted instead of ILEX and represent a cheaper and more practical alternative.

## 2. Materials and Methods

### 2.1. Research Design

A cross sectional study design was adopted with one asymptomatic control group and one chronic LBP group, in order to investigate whether a correlation exists between the two methods of testing (TEX absolute endurance and ILEX strength). The study was approved by the Centre for Health, Exercise and Sport Science ethics committee at the first author’s institution, and was conducted within the Sport Science Laboratories. Prior to testing, all subjects were provided with a participant information sheet, detailing what would be asked of them as well as their right to withdraw and were then required to sign an informed consent form.

### 2.2. Participants

Thirty eight asymptomatic participants (23 males and 15 females) and 19 participants with non-specific chronic LBP (10 males and 9 females) aged between 19 and 57 years were recruited. The participants in this study were staff or undergraduate students studying at a UK higher education institution. This was a sample of convenience, with participants being recruited via email, adverts, social media and word of mouth. Inclusion criteria for participants with chronic LBP were as follows: lumbar or lumbosacral pain occurring almost daily for at least twelve weeks [46], and no medical conditions for which a maximal effort test is contraindicated. Exclusion criteria for the asymptomatic control group was back pain exceeding one week in the preceding year. General exclusion criteria were: pregnancy, sciatica, pain radiating below the knee, disc herniation, vertebral fractures, other major structural abnormalities [47] and surgery of the pelvis or spinal column. All chronic LBP participants received physiotherapist/chiropractic consultation to confirm suitability, as well as referral, prior to inclusion.

### 2.3. Instrumentation

**Figure 1:**
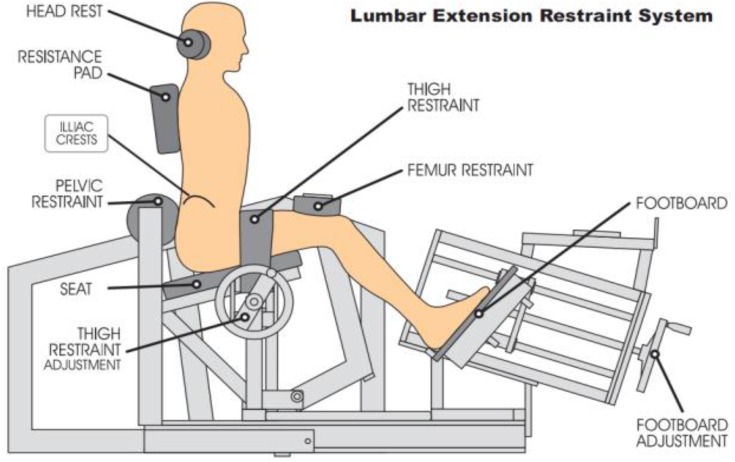
MedX schematic demonstrating the restraint system, thus isolating lumbar extensors [44].

**Figure 2:**
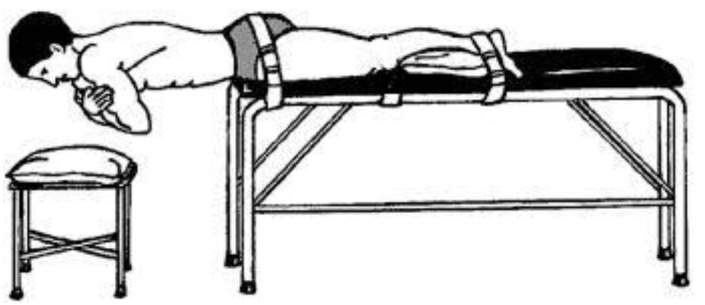
Biering-Sorensen schematic demonstrating body position and restraining belts [52].

Stature was measured using a stadiometer (Holtan ltd, Crymych, Dyfed, UK) and body mass was measured using scales (Seca, Hamburg, Germany), from this body mass index was calculated. Age, mass, stature and body mass index were similar in both asymptomatic and symptomatic participants (Table 1). Isometric strength testing for ILEX was performed using the MedX Lumbar Extension Machine (MedX, Ocala, FL, USA; Figure 1). This equipment has been found to be highly reliable through a 72 degree range of motion of lumbar extension in asymptomatic participants (*r* = 0.81–0.97; [31]) and LBP symptomatic participants (*r* = 0.57–0.93; [48]). TEX endurance was measured using the Biering–Sorensen test (Figure 2) and has been shown to produce reliable results when testing asymptomatic (ICC, 0.83) and symptomatic participants (ICC, 0.88; [49]). The Oswestry Disability Index (ODI) version 2.0 was used to assess disability and has been shown to be a valid and rigorous measure of condition-specific disability [50]. A 100-mm Visual Analogue Scale (VAS) was used to measure pain rating in chronic LBP participants [51].

### 2.4. Procedures

A Physical Activity Readiness Questionnaire (PARQ) was completed to screen for contraindications and confirm suitability based on inclusion and exclusion criteria. The participants visited the laboratory for testing on two separate occasions. These test days were separated by at least 72 h to allow the participants to recover from any residual fatigue or soreness that might have been associated with the testing [31]. The first testing day included the collection of anthropometric data, followed by TEX using the Biering-Sorensen and finally, a familiarisation session for ILEX testing. The Biering-Sorensen test was performed according to the following prescription. The participant was positioned prone on a treatment couch with the upper edge of the iliac crests aligned with the edge of the couch. The lower body was fixed to the couch by two straps, located at the level of the greater trochanter of the femur and at the ankles as close to the malleoli as possible [50]. The straps were tightened securely, whilst causing minimal discomfort to the participant. Whilst the participants were secured into position they were allowed to rest their upper body on a stool for comfort and to minimise fatigue. At the start of the test, the participants placed their arms diagonally across their chest and maintained a neutral position for as long as possible. The time the position could be held was measured using a stopwatch. Termination of the test occurred as follows: excessive fatigue, downward sloping of the trunk by more than 10° (as observed by visual inspection), unendurable pain or when 240 s was reached [52]. If the participant’s horizontal position dropped, they were asked to regain horizontal alignment until it could no longer be successfully performed. Participants were verbally encouraged to hold the position for as long as possible.

The familiarisation session on the MedX was performed as follows in order to produce reliable results [31]. The participants were seated in the lumbar extension machine, with their thighs parallel to the seat and their toes slightly inverted. A thigh restraint was placed over the lap and tightened securely, limiting movement at the thigh and pelvis. A femur restraint was placed above the flexed knees and the feet were pressed against the foot boards, which were then tightened securely. This drives the femurs towards the pelvis, thus securing the pelvis against the lumbar pad. Tests were carried out to ensure there was limited rotation of the lumbar pad and limited movement at the femur restraints; this ensures isolation of the lumbar extensors. The headrest was adjusted to the level of the occipital bone for comfort, support and positional standardisation [31]. Participants were asked to maintain a light grip on the handles during testing procedures to maintain standardisation.

After the participant had been seated, initial testing was carried out to check for any limitations in their range of lumbar motion between 0° and 72° of flexion and to adjust the counterweight to neutralise the gravitational forces of head, torso and upper extremities [31]. A slow, controlled dynamic warm-up was administered, lasting for approximately one minute. For the maximal test, the movement arm was locked into place at each specified angle (0, 12, 24, 36, 48, 60, 72 degrees if full ROM was achieved) and the participant was instructed to gradually build up tension to a maximal effort over a 3 s period. The movement arm of this testing device is attached to a load cell that is interfaced to an IBM microcomputer [30], which allows for torque to be calculated. Between each isometric contraction a rest period of ten seconds was provided, whilst being rocked to relax their lumbar extensors. 

The second day of testing occurred with at least 72 h of rest following the first test day. Prior to testing, participants were required to complete the ODI and mark the VAS. Participants then followed the same protocol for the MedX that was conducted during the familiarisation session. 

### 2.5. Data Analysis

Results from the testing were analysed using the Statistical Package for the Social Sciences 22.0 software (IBM, Portsmouth, Hampshire, UK), with an alpha level of 0.05 set as the level of statistical significance. The Shapiro-Wilk test was used to examine assumptions of normality of distribution as research has shown it to be the most powerful test for all types of distributions and sample sizes [53]. Following the Shapiro-Wilk test, demographic data was examined for between group difference using an independent *t*-test for normally distributed data and a Mann-Whitney U for data which was not normally distributed. Since the data were found to be normally distributed, a Pearson’s correlation was calculated to analyse whether there was an association between ILEX and TEX for both the combined sample and the asymptomatic and chronic LBP groups individually. Correlations were also run with additional sub-grouping for sex however results did not differ from the pooled sex analyses and so only the pooled results are reported. Correlation coefficients were interpreted as low (*r* = 0.30 to 0.50), moderate (*r* = 0.50 to 0.70) or high (*r* > 0.70) [54]. TEX endurance is reported as Biering-Sorensen hold time (BSHT) and ILEX as a strength index (SI) which was calculated as the area under the strength curve by the MedX software using the trapezoidal method, incorporating isometric strength at all tested angles.

## 3. Results

### 3.1. Participants

Participant demographics are shown in Table 1. An independent *t*-test was conducted on the normally distributed data, which revealed no significant differences between the groups for any of the variables (stature, mass and blood pressure). Age, BMI and ROM were not normally distributed and so a Mann Whitney U test was carried out on these variables. This test also revealed no significant differences between the two groups. The results from the ODI classified the CLBP participants as having only moderate disability, which may explain the lack of difference in lumbar ROM.

### 3.2. Correlations between BSHT and SI

**Figure 3:**
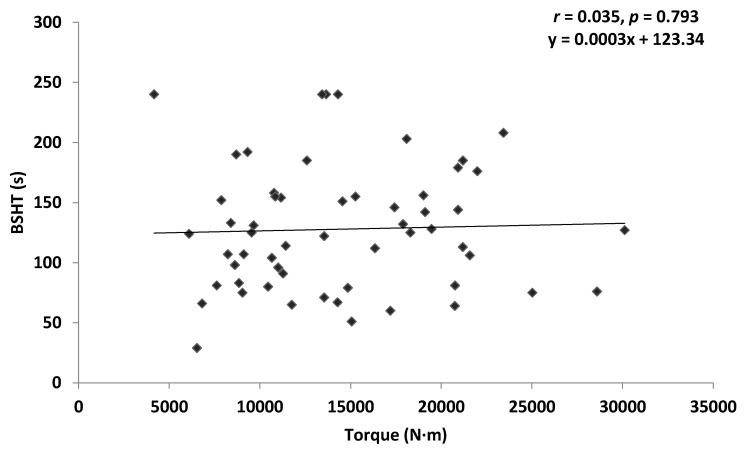
Scatter plot of combined group data for isolated lumbar extension (ILEX) strength index (SI) and trunk extension (TEX) endurance (Biering-Sorensen hold time (BSHT)).

**Figure 4:**
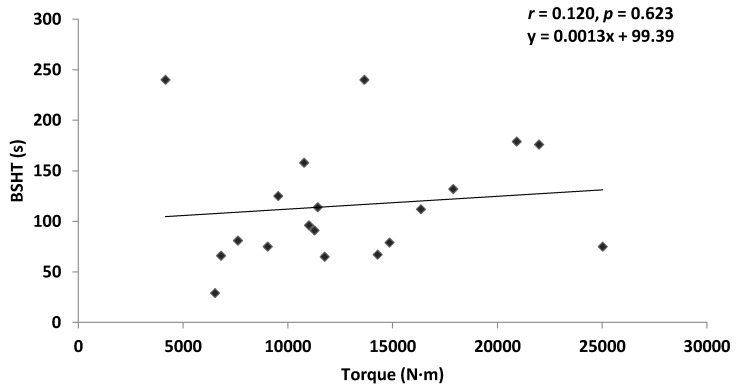
Scatter plot of chronic lower back pain (CLBP) data for ILEX strength (SI) and TEX endurance (BSHT).

**Figure 5:**
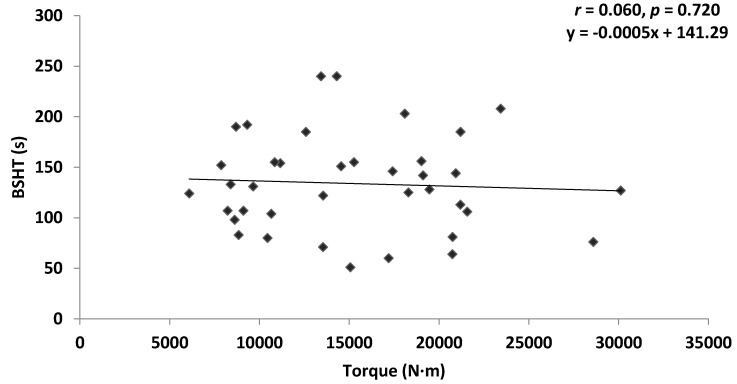
Scatter plot of asymptomatic data for ILEX strength (SI) and TEX endurance (BSHT).

Pearson’s correlation revealed a non-significant very weak positive correlation between ILEX strength and TEX endurance in the combined group (*r* = 0.035, *p* = 0.793), and the chronic LBP group (*r* = 0.120, *p* = 0.623). A non-significant very weak negative correlation was found between ILEX strength and TEX endurance in the asymptomatic group (*r* = −0.060, *p* = 0.720). Figure 3, Figure 4 and Figure 5 present scatter plots of data for ILEX strength (SI) and TEX endurance (BSHT).

## 4. Discussion

The aim of this study was to investigate whether there is a relationship between ILEX strength and TEX endurance in chronic LBP and asymptomatic participants. Statistical analysis revealed that there was no significant correlation between the two methods of testing in either the combined group (*r* = 0.035, *p* = 0.793), chronic LBP group (*r* = 0.120, *p* = 0.623) or asymptomatic group (*r* = −0.060, *p* = 0.720). This suggests that TEX endurance is a poor indicator of ILEX strength, thus supporting previous research which suggests that the pelvis must be adequately restrained and stabilised for the purpose of testing ILEX strength [31].

TEX utilises both the paraspinal muscle group and hip extensors in order to extend through a range of motion of approximately 180 degrees [30]. The hip extensors have a larger cross-sectional area and longer moment arms compared to the small lumbar extensors [55]. Further, there is a de-recruitment of the lumbar extensors and a further increase in hip extensor muscle activity during the Biering-Sorensen test [56]. Thus, the torque contributed to TEX by the hip extensors is comparatively greater than the paraspinal musculature [57]. Though, the relative contribution of the hip extensors has produced considerable discourse where some researchers suggest loading activates mostly the lumbar extensors [58,59], whereas others suggest the test indicates more about the endurance of the hip extensors [60,61]. Nonetheless, it is clear that the hip extensor musculature has the potential to influence tests of TEX endurance. Deconditioning in LBP does not appear to be present in the hip extensor musculature [55], which may explain why there is a poor relationship between the two methods of testing in both chronic LBP and asymptomatic participants.

Despite the relationship between strength and absolute endurance [33], the data presented supports previous research suggesting there is a poor relationship between tests of TEX endurance and ILEX strength [62]. As the Biering-Sorensen test utilises both the lumbar extensors and hip extensors, endurance times are not specifically indicative of the lumbar extensors [63]. Therefore, studies implementing an ILEX approach allow a more specific indication of the effects of LBP on the lumbar extensors. This is likely a result of the pelvic stabilisation preventing hip extensor contribution, and isolating the lower spine for ILEX testing [32]. At most, only 3 degrees of pelvic rotation occurs during ILEX, likely as a result of soft tissue compliance. This, in turn, results in greater reliability of results [63].

The present data suggests that ILEX strength testing is required to provide valid information regarding the function of the lumbar extensor musculature specifically. This is particularly important when examining the effects of LBP since atrophy of the lumbar extensors has been well-documented in the literature [55,64,65], potentially resulting in impaired ILEX strength [26,41,42,43,45,46]. Despite this, consideration of both tests of TEX and ILEX might be valuable when considered together [66]. In combination, the testing might allow for identification of the weak link within the posterior kinetic chain. As a result, an identification of whether differences predominantly lie between the lumbar extensors or hip extensors can be achieved. Therefore, future research examining the relationships between LBP and lumbo-pelvic function should implement a combination of both TEX endurance and ILEX strength tests. Alternatively, it may be that modifications of the Biering-Sorenson test to examine TEX endurance may have greater association with ILEX strength than the one examined here [67].

### Limitations

One limitation within this study is the varying group sizes, with a notably smaller sample size for the CLBP group. The sample of subjects may not truly represent the heterogeneity of patients with non-specific CLBP. For instance, results from the ODI classified the CLBP participants as having only moderate disability. As a consequence, this particular sample set may not have been significantly impaired, thus potentially limiting the external validity of the study. Considering this further, the degree to which psychosocial factors, kinesiophobia, or catastraphisation was present in our sample was not investigated and so whether this may have affected the results is unknown.

Intra- and inter-examiner reproducibility tests were not performed prior to the study. Though the reproducibility of TEX and ILEX tests has been previously reported to be moderate to high, this could be considered a potentially limiting factor. Further to this, only ILEX was preceded by a familiarisation with this test. Justifiably, a second session on the MedX was implemented as previous analysis revealed a significant learning effect [31] whereas the Biering-Sorensen test has previously demonstrated no learning effects [68]. However, it may have been appropriate to precede both ILEX and TEX with a familiarisation session. Lastly, for practicality and to represent application in clinical practice, visual inspection was used for the Biering-Sorenson test potentially affecting the validity of the measure in addition to an upper threshold of 240 s. This may have limited the extent to which a relationship was present in those with greater TEX endurance.

## 5. Conclusions

The present study found that there is a poor relationship between measures of TEX endurance and ILEX strength in both those with and without LBP. Research supports that persons suffering from LBP display atrophy of the lumbar musculature [55,64,65]. Since the lumbar extensors play a significant role in conditions such as LBP [63] it is important that testing mechanisms are able to elucidate which aspects of the musculature are responsible. The poor relationship between TEX endurance and ILEX strength suggests that if information regarding the function of the lumbar extensors is of interest this is best obtained by conducting ILEX testing. Results from TEX tests should only be considered as representing TEX as a compound movement and should not be used to infer results regarding lumbar extensor function specifically. However, a combination of both TEX and ILEX tests offers the greatest and most comprehensive information, by identifying whether differences predominantly lie between the lumbar extensors or hip extensors.

## Figures and Tables

**Table 1:** Participant demographics and descriptive statistics.

Characteristic	Chronic LBP (*n* = 19)	Asymptomatic (*n* = 38)	*p*-Values
**Age (year)**	28 ± 12	31 ± 12	0.218
**Stature (cm)**	173.00 ± 0.10	174.00 ± 0.10	0.642
**Mass (kg)**	75.03 ± 13.15	75.4 ± 12.60	0.918
**BMI (kg/m^2^)**	25.00 ± 3.17	24.82 ± 3.54	0.785
**SBP (mmHg)**	133.32 ± 13.97	134.47 ± 13.47	0.764
**DBP (mmHg)**	75.37 ± 10.25	73.90 ± 9.69	0.597
**Lumbar ROM (°)**	68.05 ± 6.33	68.26 ± 5.25	0.940
**VAS (mm)**	34.84 ± 24.45	NA	NA
**ODI (%)**	23.37 ± 12.33	NA	NA

Results are mean ± SD. BMI: Body Mass Index; VAS: Visual Analogue Scale; ODI: Oswestry Disability Index; NA: Not applicable.
